# A Cross-Sectional Study of Time Since Death From Image Analysis of Corneal Opacity

**DOI:** 10.7759/cureus.14975

**Published:** 2021-05-11

**Authors:** Anamika Nath, Amar J Patowary, Amarantha Donna Ropmay, Daunipaia Slong, Kumar Pinku Pratim, Bashan Kupar Rymbai, Rangme Betlin Yvette Marbaniang

**Affiliations:** 1 Department of Forensic Medicine, North Eastern Indira Gandhi Regional Institute of Health & Medical Sciences (NEIGRIHMS), Shillong, IND; 2 Department of Forensic Medicine, Gauhati Medical College & Hospital, Guwahati, IND

**Keywords:** time since death, corneal colour, rgb software

## Abstract

Estimation of time since death (TSD) is an important aspect of forensic medicine. Methods used so far are subjective and have human errors. Corneal opacity images using software to analyze the red, green, and blue (RGB) components of corneal color against the TSD may prove to be an objective method. This study aimed to estimate TSD from image analysis of corneal opacity from the cornea of deceased individuals brought in for medicolegal autopsy to study the factors affecting corneal opacity and to formulate a predictive equation for the estimation of TSD. This was a cross-sectional study conducted at the department of forensic medicine and toxicology of a tertiary care medical institute over two years. The study group included cadavers brought in for autopsy where the TSD was known from hospital records. For study tools, we used a digital single-lens reflex (DSLR) camera with standardized settings, a dark box made of cardboard, and open-access RGB analysis software. Images were analyzed for differences in the numeric values of the RGB color and compared against the TSD. Correlations between TSD and age, gender, and environmental temperature were checked. This study involved 30 cases; these were analyzed and showed an increase in the numeric values of RGB for the corneal color as the TSD increases. Of note, the correlation of TSD with the color red was greater than for either blue or green; age had a positive correlation while gender had nearly no correlation, and the environmental temperature had a negative correlation. Based on this, gender was excluded from our equation. Also, we noted that the variance inflation factor of green was high and, therefore, excluded it from the predictive equation. The equation derived follows: TSD = {(0.091 x Age) + (0.171 x Red) + (0.018 x Blue) - (0.019 x Environmental Temperature) - 5.263}. Using this equation, the mean error was 21 minutes. This equation further narrowed the time range, usually given as four to six hours, when determining the TSD via conventional methods. Image analysis of corneal color after death using RGB analysis software can give us a more accurate and human error-free TSD that can be digitally stored and reproduced and, therefore, could prove useful in the forensic arena in the future.

## Introduction

The main objectives of an autopsy include finding out the cause of death, time since death (TSD), manner of death, mode of death, and the like. Estimation of the TSD is a necessary feature of forensic medicine. Corneal opacity increases with TSD and has been routinely used to estimate TSD [1]. However, currently, the temperature of the body, rigor mortis, and post-mortem hypostasis are given precedence when calculating TSD, and over time, the importance of corneal opacity is declining amongst autopsy surgeons/autopsy pathologist. The main reason attributed to this is that the progression of corneal opacity has not been standardized against the TSD. Earlier methods that were used to calculate TSD, on the basis of the temperature of the body, rigor mortis, and post mortem staining, are subjective, and, as such, human error is inevitable. Therefore, there is a need for a more accurate and objective method that is reproducible and from which the data can be stored. Also, the TSD is often given in the range of four to six hours; this may not help the investigating agencies. Therefore, it is of utmost importance to devise a method that can overcome such lacunae. To establish an objective, accurate, reproducible, and human-error-free method for the estimation of TSD, we used RGB (Red, Green, Blue) image analysis of the corneal color of the deceased using open-source software freely available on the internet (Image J: https://imagej.nih.gov/ij/index.html). The aim of this study was to estimate TSD from image analysis of corneal opacity from the cornea of deceased persons brought for autopsy and to study the factors affecting corneal opacity. In addition, we wanted to formulate a predictive equation for the estimation of TSD by which TSD could be calculated in the future from corneal images. Hence, we hope this study may help in restoring the value of analyzing corneal opacity in relation to TSD. This is the first of its kind research in India and would make autopsy surgeons'/autopsy pathologists' work easier, more accurate, and technology-based. There is no invasive procedure used, and hence the aesthetic appearance of the eyes is not disturbed. The method described here is objective, easy to use, and TSD can be estimated within a very short period of time, potentially making this method particularly useful. To further increase the accuracy of TSD estimation, other quantifiable parameters were also evaluated.

## Materials and methods

**Figure 1:**
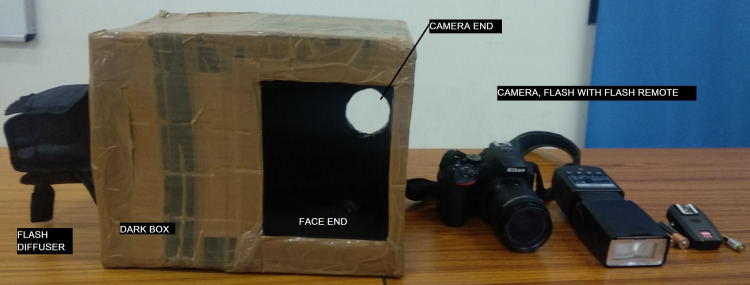
Study tools used

The study design is cross-sectional in nature and was performed at the Department of Forensic Medicine and Toxicology of a tertiary care medical institute over a period of two years, from October 2018 to October 2020. Permission for this study was obtained from the institution's ethics committee. The study group was cadavers brought for medicolegal autopsy in which the time of death was known from hospital records. Those cadavers, with obvious defects of the cornea or with burns, injuries, or decomposition, were excluded from the study. The study tools included one digital single-lens reflex (DSLR) camera with standard settings, a dark box made of cardboard, and open-access RGB analysis software. Using the software, images were evaluated to obtain numeric values for RGB and compared against TSD. The numeric values for each shade of red, green, and blue ranged from 0-255 in the software. Both eyes of the cadavers were photographed using a Nikon D5600 with standardized manual settings of ISO 200, shutter speed 100, and a focal length of 5.6. A dark box measuring 30 cm x 25 cm x 20 cm with two holes in opposite parallel surfaces was made from cardboard. Through one, the camera was introduced while the other hole was placed above the eye to be photographed. The distance between the camera and eye was fixed for all the cases at 25 cm, the minimum distance required for photographing with a kit lens (18-55 mm). An external flash was used from the top of the box, through a diffuser, and remotely operated through the camera. The external electronic flash used was a model DFL-077 from Digitek (New Delhi, India) (Figure 1).

**Figure 2:**
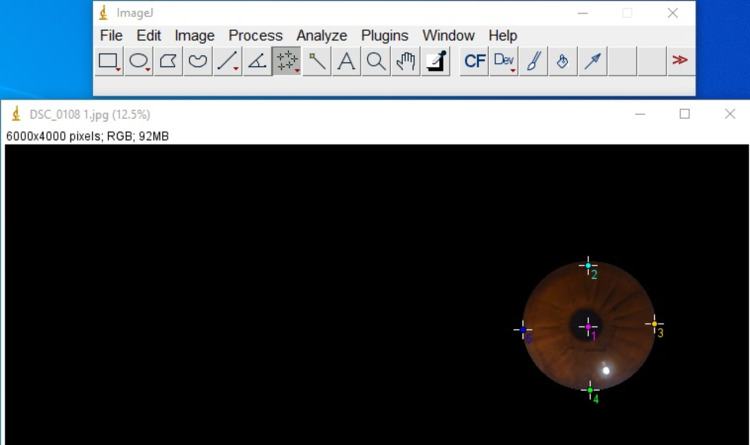
Selection of points on the cornea for determining their RGB value

All the photographs were then entered into the open-access software, Image J, which assigns each shade of a color (red, green, and blue) a different numeric value. The corneal image was enlarged 2x. Five points from the image on the nearly circular cornea were selected using x and y coordinates. The left, right, top, bottom, and center points were marked, and each of their red, green, and blue numerical values were noted and the average calculated (Figure 2). Similarly, the mean of the left and right eye values was noted for these three colors. The original TSD was calculated as the difference between the time of death from hospital records and the time of examination. All the values were entered into the Statistical Package for the Social Sciences (SPSS) software, version 21.0 (Released 2012, IBM Corp, Armonk, NY), and Microsoft Excel version 2007 (Microsoft Corporation, Redmond, WA). These variables were the time of death, age, and gender from hospital records, the environmental temperature at the conduction of autopsy, and the average values for red, green, and blue from both eyes. TSD was evaluated and designated as the dependent variable. Age, gender, environmental temperature, and average values of red, green, and blue were designated as independent variables. The bivariate correlation between TSD and each of the independent variables was analyzed. Scatter plots were made, and the correlations were further evaluated. Pearson correlation was used to measure each of the variables against TSD. A P-value of < .05 was considered to be statistically significant. The effects of all these variables on TSD were assessed through a multiple regression model. With the help of a variance inflation factor, each variable was assessed for multi-collinearity before conducting a multiple regression analysis and formulating the equation. Analysis of covariance (ANCOVA) was tested between the independent variables. A predictive equation was hence formulated. The TSD calculated from the equation was compared to the original TSD, and the corresponding mean error was recorded.

## Results

**Figure 3:**
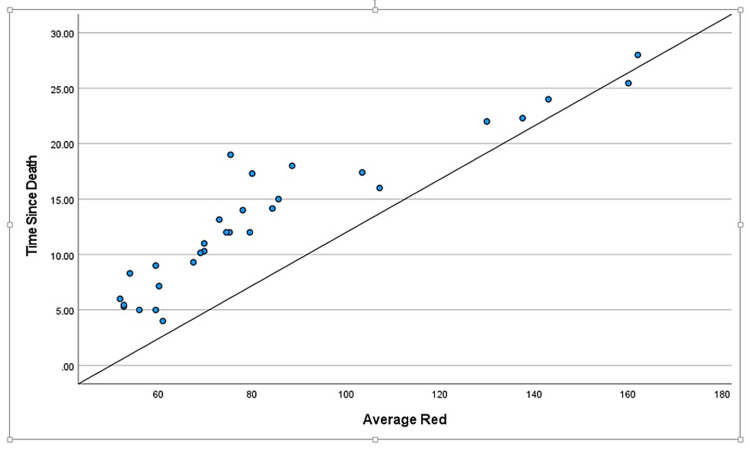
Scattered plot between TSD and red Abbreviation: TSD, time since death

**Figure 4:**
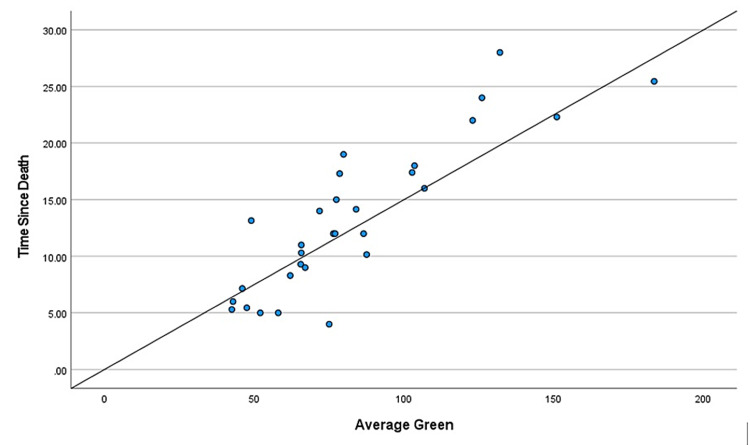
Scattered plot between TSD and green Abbreviation: TSD, time since death

**Figure 5:**
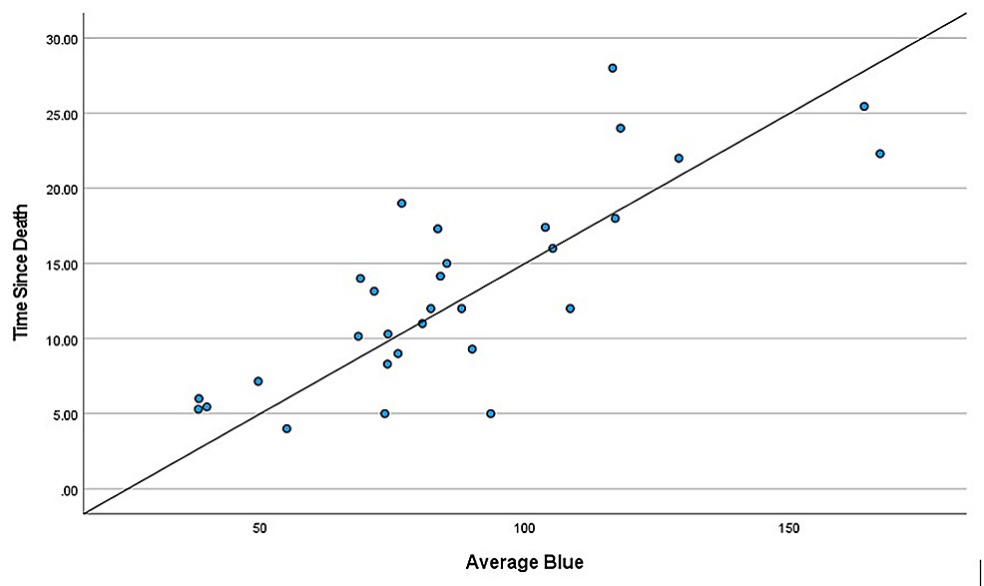
Scattered plot between TSD and blue Abbreviation: TSD, time since death

**Figure 6:**
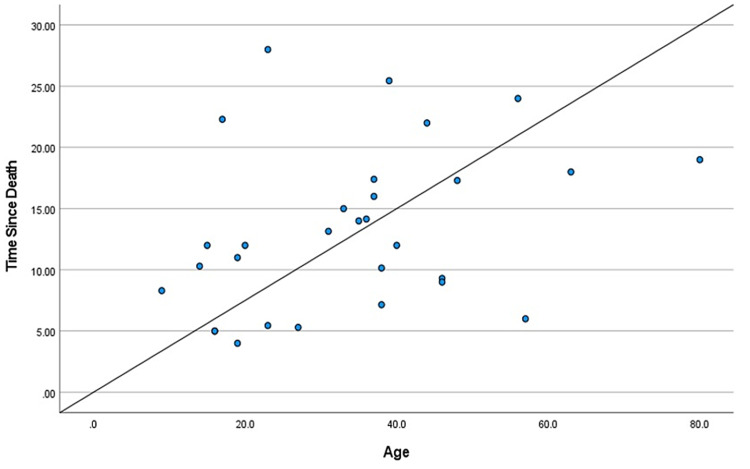
Scattered plot between TSD and age Abbreviation: TSD, time since death

**Figure 7:**
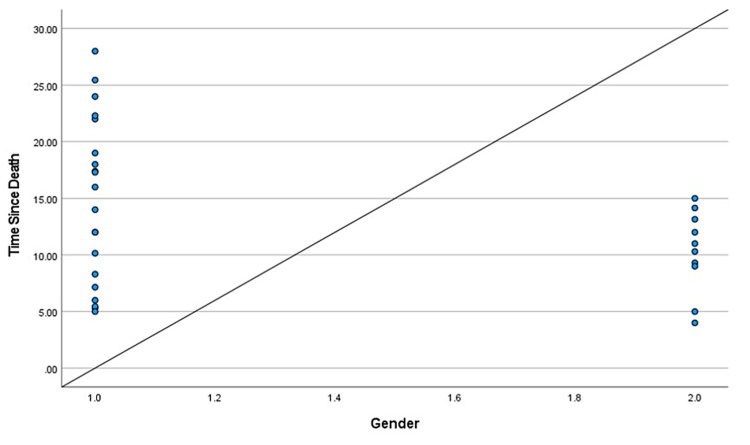
Scattered plot between TSD and gender Abbreviation: TSD, time since death

**Figure 8:**
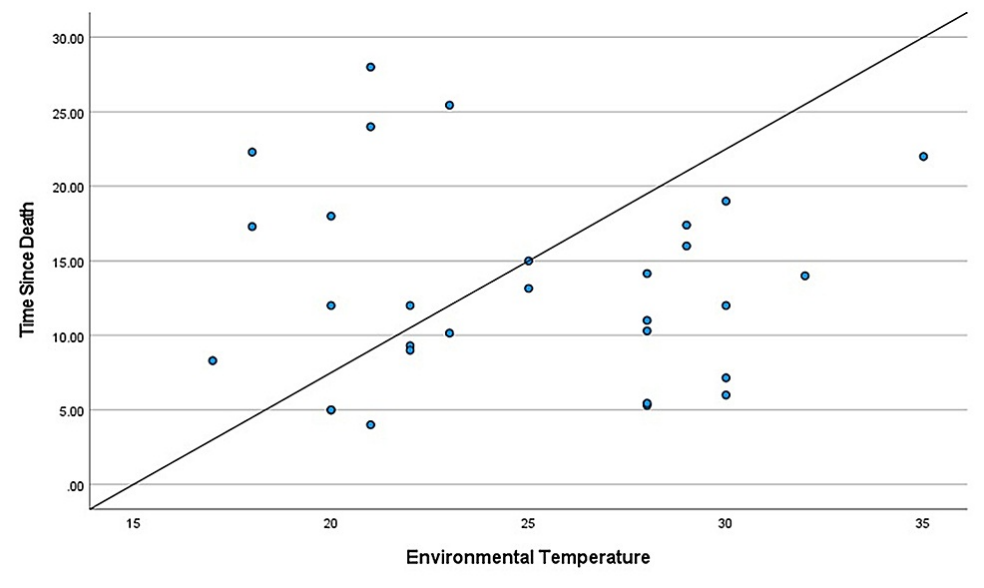
Scattered plot between TSD and environmental temperature Abbreviation: TSD, time since death

During the two-year study, a total of 114 cases came in for medicolegal autopsies in our department. However, only 30 cases were included in this study as they had hospital records providing the time of death. The rest were excluded due to injuries to the craniofacial area, burns, and decomposition, and the majority because the bodies came from outside the hospital with no record of the time of death. Those that were kept in the freezer during the previous night were excluded from the study as freezing delays the progression of corneal opacity. The mean age of the deceased subjects was found to be 34.06 years, the age range from nine to 63 years. We considered cases within 48 hours of the time of death to avoid cases with changes due to decomposition. The mean TSD was found to be 13.25 hours, the range of time from five to 25.45 hours. There were positive correlations between TSD and color changes in each of the basic colors: red, green, and blue (Table 1, Table 2, and Table 3, respectively). The different shades of these three basic colors carried specific numeric values in the RGB software. It was found that these numeric values increased with intensified colors or progressing corneal opacity, as well as with an increase in TSD. Positive correlations were found between TSD and age, and a negative correlation was found between TSD and environmental temperature (Table 4 and Table 5, respectively). Gender had no specific correlation with TSD and was excluded from the study. Scatter plots were further made for each of the bivariate correlations (dependent on each independent variable), and the factors affecting TSD were determined for red, green, blue, age, gender, environmental temperature (Figure 3, Figure 4, Figure 5, Figure 6, Figure 7, Figure 8). The correlations between TSD and the RGB colors, age, and environmental temperature each had P values of < .05 and hence were statistically significant. The color green had a variance inflation factor of more than 10 and a tolerance of 0.071 and hence was excluded from the regression analysis (Table 6). We checked the tests of between-subject effects through ANCOVA and found no significance or correlations between the independent variables. Hence, we included all of them in the regression model except the color green and gender.

**Table 1:** Correlation of time since death with average red value Abbreviation: Sig, significance; TSD, time since death

		TSD	Average Red
TSD	Pearson Correlation	1	.930^**^
	Sig. (2-tailed)		.000
	n	30	30
Average Red	Pearson Correlation	.930^**^	1
	Sig. (2-tailed)	.000	
	n	30	30

**Table 2:** Correlation of time since death with average green value Abbreviation: Sig, significance; TSD, time since death

		TSD	Average Green
TSD	Pearson Correlation	1	.866^**^
	Sig. (2-tailed)		.000
	n	30	30
Average Green	Pearson Correlation	.866^**^	1
	Sig. (2-tailed)	.000	
	n	30	30

**Table 3:** Correlation of time since death with average blue value Abbreviation: Sig, significance; TSD, time since death.

Correlations			
		TSD	Average Blue
TSD	Pearson Correlation	1	.800^**^
	Sig. (2-tailed)		.000
	n	30	30
Average Blue	Pearson Correlation	.800^**^	1
	Sig. (2-tailed)	.000	
	n	30	30

**Table 4:** Correlation of time since death with age Abbreviation: Sig, significance; TSD, time since death

		TSD	Age
TSD	Pearson Correlation	1	.339
	Sig. (2-tailed)		.066
	n	30	30
Age	Pearson Correlation	.339	1
	Sig. (2-tailed)	.066	
	n	30	30

**Table 5:** Correlation of time since death with environmental temperature Abbreviation: Sig, significance; TSD, time since death

		TSD	Environmental Temperature
TSD	Pearson Correlation	1	-.028
	Sig. (2-tailed)		.882
	n	30	30
Environmental Temperature	Pearson Correlation	-.028	1
	Sig. (2-tailed)	.882	
	n	30	30

**Table 6:** Determination of coefficients and variance inflation factor of the variables Abbreviation: Sig, significance; TSD, time since death; VIF, variance inflation factor

Model		Unstandardized Coefficients		Standardized Coefficients	t	Sig.	95.0% Confidence Interval for B		Collinearity Statistics	
		B	Std. Error	Beta			Lower Bound	Upper Bound	Tolerance	VIF
1	(Constant)	-5.091	3.117		-1.633	.116	-11.539	1.357		
	Average Red	.200	.036	.967	5.485	.000	.125	.275	.115	8.719
	Average Green	-.051	.044	-.258	-1.156	.259	-.142	.040	.071	13.989
	Average Blue	.040	.033	.194	1.236	.229	-.027	.108	.145	6.891
	Age	.096	.026	.240	3.690	.001	.042	.149	.844	1.184
	Gender	-.247	.888	-.018	-.278	.783	-2.084	1.590	.856	1.168
	Environmental Temperature	-.028	.095	-.020	-.292	.773	-.224	.169	.745	1.343

The predictive equation we formulated is as below:

TSD = {(0.091 x Age) + (0.171 x Red) + (0.018 x Blue) - (0.019 x Environmental Temperature) - 5.263}

Following this equation, predictive values were calculated for these 30 data sets, and the standard deviation was found to be 1.917. Mean error was found to be +/- 0.35 hours or 21 minutes.

## Discussion

In forensic literature, post-mortem eye changes are usually discussed. All post-mortem ocular changes are classified as early post-mortem changes [2]. Loss of corneal reflex, clouding of the cornea, and the so-called "tache noir" (wrinkled and brown corneal surface exposed between eyelids) are some of the post-mortem corneal changes [3]. Pupils react to atropine for about an hour after death [4]. This simple test cannot be performed routinely as all the bodies are brought for post-mortem examination after a minimum of two hours of death. Previously, the grayscale method was used, which estimated TSD through comparison of the degree of corneal opacity to the grayscale, but this method also relies on the autopsy surgeons' judgment and is variable. This was based on the turbidity of the cornea [4]. Apart from this, many other objective methods for the calculation of TSD were developed by research workers, but they are invasive in nature and require removal of eyeballs/cornea, which affects the appearance of the cadavers and is typically not acceptable to their family members. These methods include the measurement of laser light transmittance [5-6]. Some other research included measuring corneal thickness for the estimation of TSD [7]. In some studies, calculation of the number of dead endothelial cells in the cornea was advocated as an estimation of TSD [8-9]. Another study promoted the estimation of the state of DNA degradation in the corneal cells in order to calculate the TSD [10]. These methods require special skills and procedures and are time-consuming. Additionally, the majority of them were invasive, and, as such, a newer method would be beneficial.

To this end, image analysis is a newer method for TSD estimation. Previous researchers like Liu et al. had used corneal image analysis on mice [11], and Zhou et al. had tried the same procedure on the corneas of rabbits [12]. During their research, they found a correlation between corneal opacity and TSD through image analysis and noted that it increased with an increase in TSD. Kawashima et al. had studied image analysis of human cornea and found a positive correlation between corneal color, as estimated from RGB shading, and actual TSD for both room light and LED light sources [13]. Of note, Kawashima et al. observed differences while photographing the cornea by room light and by LED. In our study, we eliminated this factor by photographing in a dark box. Also, in the study by Kawashima et al., the camera settings were not standardized, and, as such, the corneal color may have been perceived differently due to different settings of the camera at the same point of time after death.

Our study emphasizes the utility of computer-based analysis of TSD. The corneal color, as demonstrated by shades of RGB, intensified with TSD, as did corneal opacity. We modified the methodology as specified by Kawashima et al. and conducted it in a setting and population center located in India. To further improve the objectivity of the method, we used a standardized software-based image analysis methodology for evaluating the intensity of the shades of RGB via their numerical value for each shade ranging from 0-255. Also, the data points on the cornea are specific due to standardized x and y coordinates.

This study proposes a reliable, human error-free, fast, non-invasive procedure for determining the TSD that is reproducible and, as it is digital, can be stored for years to come. We developed an equation based on our analysis and found the mean error to be 0.35 hours or 21 minutes; this error range is acceptable since we usually give the TSD in a range of four to six hours; therefore, this method could narrow the range for TSD. However, since the data only included 30 individuals, the equation should be tested on more subjects in order to generalize and further validate the equation.

## Conclusions

The analysis of images of corneal opacity after death using software to quantify RGB values can give us a more accurate and human error-free TSD, which can be stored digitally. Our results demonstrate that, in a small sample, easily obtained absolute values for corneal colors (red, blue) can be analyzed digitally and combined with the individual's age and the environmental temperature of the body to provide a more accurate determination of TSD. This can be efficiently applied in a short span of time and could prove to be useful in the forensic specialty in the years to come.
